# *PTB-XL+*, a comprehensive electrocardiographic feature dataset

**DOI:** 10.1038/s41597-023-02153-8

**Published:** 2023-05-13

**Authors:** Nils Strodthoff, Temesgen Mehari, Claudia Nagel, Philip J. Aston, Ashish Sundar, Claus Graff, Jørgen K. Kanters, Wilhelm Haverkamp, Olaf Dössel, Axel Loewe, Markus Bär, Tobias Schaeffter

**Affiliations:** 1 Oldenburg University, Oldenburg, Germany; 2 Physikalisch-Technische Bundesanstalt, Berlin, Germany; 3 Fraunhofer Heinrich Hertz Institute, Berlin, Germany; 4 Karlsruhe Institute of Technology, Karlsruhe, Germany; 5 National Physical Laboratory, Teddington, UK; 6 University of Surrey, Guildford, UK; 7 Aalborg University, Aalborg, Denmark; 8 University of Copenhagen, Copenhagen, Denmark; 9 Charité - Universitätsmedizin, Berlin, Germany; 10 TU Berlin, Berlin, Germany; 11 King’s College London, London, UK

**Keywords:** Cardiovascular diseases, Cardiovascular diseases

## Abstract

Machine learning (ML) methods for the analysis of electrocardiography (ECG) data are gaining importance, substantially supported by the release of large public datasets. However, these current datasets miss important derived descriptors such as ECG features that have been devised in the past hundred years and still form the basis of most automatic ECG analysis algorithms and are critical for cardiologists’ decision processes. ECG features are available from sophisticated commercial software but are not accessible to the general public. To alleviate this issue, we add ECG features from two leading commercial algorithms and an open-source implementation supplemented by a set of automatic diagnostic statements from a commercial ECG analysis software in preprocessed format. This allows the comparison of ML models trained on clinically versus automatically generated label sets. We provide an extensive technical validation of features and diagnostic statements for ML applications. We believe this release crucially enhances the usability of the *PTB-XL* dataset as a reference dataset for ML methods in the context of ECG data.

## Background & Summary

Cardiovascular diseases continue to be one of the largest burdens for the population worldwide^1^. Due to its simplicity, non-invasive nature, widespread use and diagnostic value, the electrocardiogram (ECG) is one of the primary tools for the first assessment. However, it requires the analysis of a huge amount of time-series ECG-data. Therefore automatic analysis tools have become standard. The recent developments in machine learning/AI have demonstrated its potential in this direction^2–5^. Large freely available ECG databases^6,7^ are crucial for the development and benchmarking of AI algorithms for automatic classification. Consequently, they have been the basis of recent competitions and challenges^8,9^. Even though end-to-end trained deep learning models are on the rise, handcrafted features continue to play an important role in ECG analysis: They involve decades of engineering and encode valuable domain knowledge used for clinical diagnosis. Most of the ECG features are inherently interpretable for domain experts and represent a very efficient way to perform patient stratification. Furthermore, their availability allows investigating the extent to which deep models align with these features (concepts), or to directly compare to algorithms trained on manually extracted features, or potentially devise more robust algorithms relying on both. ECG features also represent a substantial reduction of the high-dimensional raw ECG time series and enable therefore comprehensive comparisons between different clinical ECG data bases. They may also be used for clinical validation of synthetic data sets stemming from simulations based on digital twins of individuals^10–12^ or virtual cohorts of realistic models^13,14^.

**Fig. 1:**
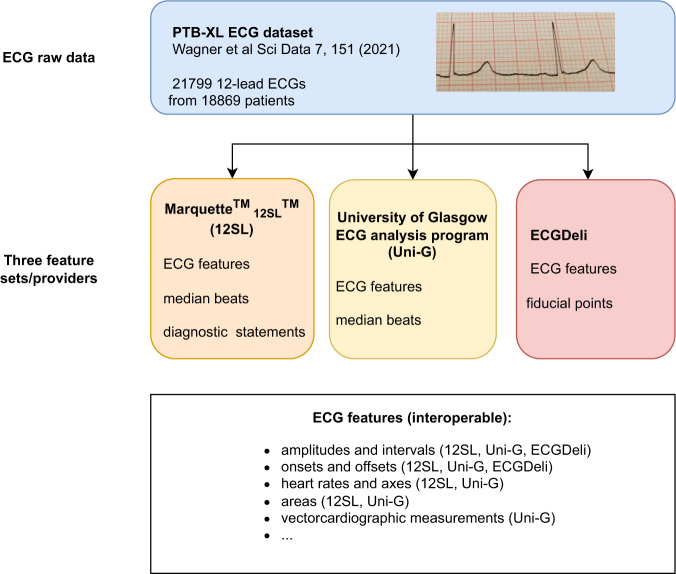
Schematic overview of the components that constitute the *PTB-XL* + dataset.

Electrocardiography is a unique domain with a long history of such handcrafted features and commercially available software packages that allow extracting them in a reliable way. However, as a practical obstacle, high-quality ECG features from commercial software are not accessible to the broader ECG research community. Furthermore, their comparative quality, also in comparison to available open-source toolkits, when applied to a comprehensive ECG dataset, is unknown. With this dataset, *PTB-XL*+, see Fig. 1 for a schematic overview, we aim to mitigate these shortcomings by releasing ECG features from two commercial and one open-source feature extractors for the entire *PTB-XL*^6,15,16^ dataset. Since its publication, the *PTB-XL* dataset quickly developed into one of the largest and most widely used publicly available 12-lead clinical ECG datasets covering a broad set of conditions with diverse signal quality and hence representative of real-world ECG data. By releasing accompanying ECG features, we hope to further strengthen the role of the *PTB-XL* dataset as a reference dataset for the development and evaluation of automatic ECG analysis algorithms. To increase the interoperability of the features from different ECG feature providers, we mapped features to a common naming scheme (including mapping to SNOMED CT^17^/LOINC^18^ ontologies) that allows using the corresponding feature sets as interchangeably as possible. Further metadata such as median beats or fiducial points further enhance the value of the dataset. Finally, the *PTB-XL* + dataset includes automatic diagnostic statements as provided by one of the most widely used commercial ECG algorithms, the Marquette 12SL (GE Healthcare, WI) algorithm. To also increase the interoperability in this respect, we provide mappings for these statements as well as for the original *PTB-XL* ECG statements to SNOMED CT statements as a common ontology and advocate this as a useful procedure to increase the interoperability of datasets that were labeled according to different ontologies. This has several important implications: First, mismatches between the 12SL statements and the original labels can be used to assess the label quality of the *PTB-XL* dataset itself. Second, it allows to directly compare the performance of models trained on the original *PTB-XL* labels provided by cardiologists to the predictions of the 12SL. The dataset was compiled with direct applicability for machine learning applications in mind and includes an extensive technical validation based on publicly available source code^19^, which can be used as a starting point for own analyses.

## Methods

### Considered algorithms

Before we describe the steps that were followed to create the *PTB-XL* + dataset, we give a brief overview of the different methodologies followed by the included ECG analysis algorithms.

### University of Glasgow ECG analysis program (Uni-G) and Marquette 12SL (12SL)

The University of Glasgow ECG Analysis Program and Marquette 12SL (GE Healthcare, WI) are two commercial, state-of-the-art ECG analysis packages that are distributed in millions of ECG devices world-wide. Both follow a similar approach: In a first step, a median/template beat is calculated. In a second step, ECG features are extracted from this median beat (in addition to some features such as heart rate that are collected from the full ECG) and in a third step diagnostic statements are predicted from these features, see^20^ for details on the Uni-G approach and^21^ for details on the 12SL algorithm. Due to usage restrictions, the *PTB-XL + *dataset includes automatic diagnostic statements only from 12SL but the full feature sets from both algorithms. Both feature extraction algorithms are closed source and only accessible on special devices or after purchase. The decision rules followed by the 12SL algorithm are available from the Physician’s Manual^21^.

### ECGDeli

ECGDeli is an open-source ECG delineation toolkit developed within the Institute of Biomedical Engineering at the Karlsruhe Institute of Technology, Germany. The feature extraction follows a different approach compared to the two approaches discussed before. It builds on the fiducial points obtained from the open-source ECGDeli^22,23^ software. ECG features are computed separately for each available beat. Even though the package is publicly available, its execution relies on MATLAB as proprietary software, which limits the range of potential users. In the dataset, we report only the median and the (0.25,0.75)-interquartile range across beats, which allows to assess the variability of features across different beats, as well as the total count of beats that were considered for each respective feature. In addition to amplitude and interval features, the dataset includes a number of morphological features.

### Data processing

The records from the *PTB-XL*^6,15,16^ dataset were converted to appropriate input formats and processed by the Uni-G, the 12SL and the ECGDeli algorithms. For 12SL, all ECGs were imported into a custom-built MUSE Cardiology Information System (GE Healthcare, Wauwatosa, WI, USA) and upon import they were reanalyzed with the latest version of 12SL (v.243). Automatic diagnostic statements were directly exported from the GE software rather than re-implemented based on the reference manual. Uni-G features were exported from a custom-built version of the Glasgow software (R30.4.2). ECGDeli features were extracted from the publicly available version 1.1 of the software.

The output features were harmonized into a unified naming scheme and converted into compatible units (using mV for amplitudes and ms for intervals as base units). However, the output features still maintain their original form as produced by each respective algorithm. The ECG features for each of the three feature sets were converted into a tabular format with a single row per ECG record and a column for each ECG feature. Additional features that were provided by the different algorithms such as fiducial points or median beats were converted to appropriate output formats and are also distributed as part of this dataset. Finally, the automatic diagnostic statements provided by 12SL were converted to a format that makes them directly applicable for training ML algorithms. Additionally, we devised a mapping both from the original *PTB-XL* statements and of the 12SL automatic diagnostic statements to SNOMED CT^17^ and applied them to the original label sets. The details are described in the following section.

## Data Records

### Data released as part of this dataset

This section describes the components of the released data repository, which is hosted by PhysioNet^16,24^. For the three feature sets, Uni-G, 12SL and ECGDeli, we provide the following collection of features:

- The **Uni-G feature set** includes ECG features and median beats from which most of the features were extracted.
- The **12SL feature set** includes ECG features and median beats from which most of the features were extracted. In addition, automatic diagnostic statements provided by the 12SL algorithm are included.
- The **ECGDeli feature set** includes median feature values across beats, corresponding (0.25,0.75)-interquartile ranges across beats and counts across beats along with the fiducial points along the rhythm strip from which the features were extracted.

Generally, we refer to ECG features as a collection of amplitudes and intervals (global as well as lead-specific), onsets of ECG segments (global as well as lead-specific), areas and similar features. The precise composition of features only depends on the availability of features in the respective algorithms. The data is organized as follows:

- ECG features (Uni-G, 12SL, ECGDeli): For each of the three feature providers, we provide feature tables as csv-files with the PTB-XL ECG identifier as key (unig_features.csv, 12sl_features.csv, ecgdeli_features.csv).

The columns follow a unified naming scheme (including mapping to SNOMED CT or LOINC where available), which allows using the three feature sets interchangeably provided the corresponding features are available in multiple datasets. A corresponding summary table (feature_description.csv) lists the available ECG features along with a short description and units of measurement. For all three feature sets, the ECG features include durations, amplitudes and on/off-sets of segments. Uni-G and 12SL include in addition area features and Uni-G also has vectorcardiographic measurements (calculated from I, aVF and V2 as quasi-orthogonal leads).

- **Fiducial points** (ECGDeli): We provide fiducial points in PhysioNet’s wfdb annotation format^25^, both lead-specific and consensus annotations across all leads. The annotations are organized in subfolders following the structure of the PTB-XL dataset with filenames relating to the PTB-XL ECG identifier.
- **Median beats** (Uni-G, 12SL): We provide median beats in PhysioNet’s wfdb signal format^25^ that can be processed analogously to the samples in the original *PTB-XL* dataset. As the fiducial points, the median beats are organized in subfolders following the structure of the PTB-XL dataset with filenames relating to the PTB-XL ECG identifier.
- **Automatic diagnostic statements** (12SL): We provide the automatic diagnostic statements as a csv-file (12sl_statements.csv) indexed by *PTB-XL* ECG identifier, where we provide both the original ECG statements assigned by the 12SL-algorithm and the statements after mapping to SNOMED CT. For every statement, we also include all parent nodes and in this way propagate the label upwards in the SNOMED CT ontology until we reach the root node of the label tree. For the user’s convenience, we provide a similar file for the statements assigned in the *PTB-XL* dataset after application of a similar mapping (ptbxl_statements.csv). We also release the tables underlying the mappings to SNOMED CT codes (12slv23ToSNOMED.csv and ptbxlMapToSNOMED.csv). In addition, we provide the code to apply a potentially modified mapping at a later point in time (apply_snomed_mapping.py). Finally, we provide a human-readable description of the used SNOMED CT concept identifiers in SNOMED_description.csv. In this table, we also mark identifiers as informative if they neither perfectly correlate with another label nor are too unspecific such as “Finding of body region”. We propose to use only this reduced set for the training and evaluation of ML algorithms, see below. Finally, we stress that we provide for the first time a way to convert automatic 12SL’s diagnostic statements into a machine-readable format that can be directly used to train machine learning models. A complete description of the available label sets in ptbxl_statements.csv and 12sl_statements.csv is given in Table 1.

**Table 1:** Description of the provided label sets.

	column	Description
12sl_statements.csv	*ecg_id*	PTB-XL ECG identifier
	*statements*	ordered list of original 12SL statements
	*statements_cat*	*statements* but with qualifier statements bound to elementary statements via semicolon; can be used to build more finegrained prediction models based on 12SL labels
	*statements_ext*	*statements_cat* separated into primary statements again keeping only AC (possibly acute) and AU (age undetermined) qualifier statements bound to elementary statements, removing WITH, AND, OR statements that cannot stand alone; (value, certainty) tuples (incorporating information from CRO (cannot rule out)/PO (possible)); default label set for prediction models based on 12SL labels
	*statements_ext_SNOMED*	*statements_ext* after mapping to SNOMED CT identifier as (value, certainty) tuples, including information from CRO/PO statements as well as uncertainties in the label mapping, with all labels propagated upwards in the SNOMED CT label hierarchy; can be used to train/evaluate models on SNOMED CT labels
ptbxl_statements.csv	*ecg_id*	PTB-XL ECG identifier
	scp_codes	original ECG statements (up to minor deviations^6^ consistent with the SCP standard^29^) as (statement, certainty) tuples, where the certainty of all non-diagnostic statements is set to 100 (as opposed to the 0 in the original dataset)
	*scp_codes_ext*	extended set of ECG statements including heart axis and information about acute/old infarction stage (where available) extracted from the *PTB-XL* metadata
	*scp_codes_ext_SNOMED*	*scp_codes_ext* after mapping to SNOMED CT identifiers as (value, certainty) tuples, with all labels propagated upwards in the SNOMED CT label hierarchy; can be used to train/evaluate models on SNOMED CT labels

### Descriptive statistics

With the exception of a small number of samples that could not be processed by particular algorithms, the feature sets cover the full *PTB-XL* dataset^6,15,16^, i.e., up to 21799 records from 18869 patients.

**Fig. 2:**
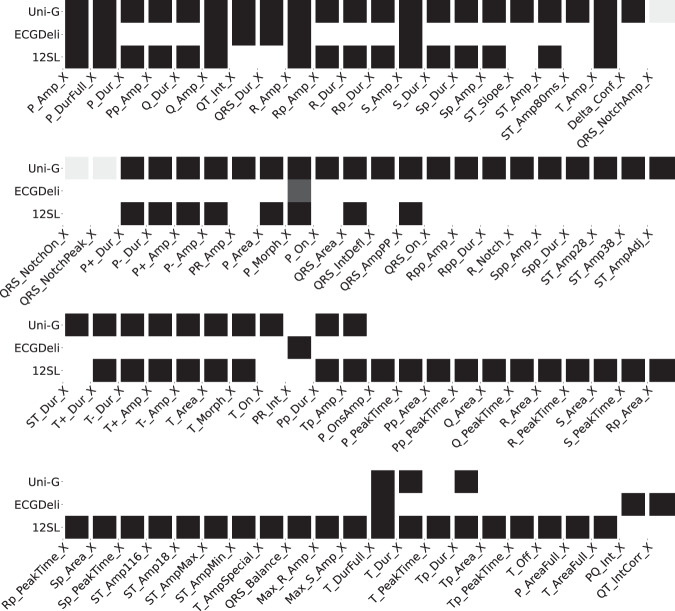
Lead- and segment-specific features as provided in the different feature sets. Color-coding corresponds to the fraction of samples for which values are present whereas black corresponds to values present for all samples. We report average statistics across leads X. The used acronyms are described in feature_description.csv.

**Fig. 3:**
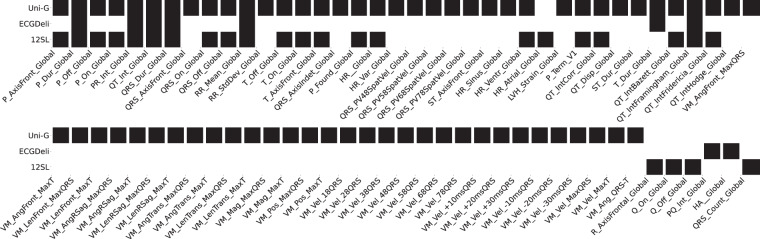
Global (sample-wise) ECG features as provided within the different feature sets. Color-coding as in Fig. 2.

We summarize the available features in each of the three feature sets in terms of two figures: Fig. 2 shows the fraction of samples in the dataset for which a particular feature is present for lead-dependent features. Figure 3 shows the analogous plot for global, i.e., lead-independent, features. The features are labeled according to their abbreviations. The corresponding descriptions can be found in feature_description.csv. Here and in the following, we use X as a placeholder for the leads, i.e., X can take values from the set {I,II,III,aVR,aVL,aVF,V1,V2,V3,V4,V5,V6}. The figures visually demonstrate that there are 13 features (counting lead-specific features only once) that are present in all three feature sets and 39 features that are present in at least two feature sets, which allows for a large number of cross-comparisons for consistency checks, see Technical Validation.

**Fig. 4:**
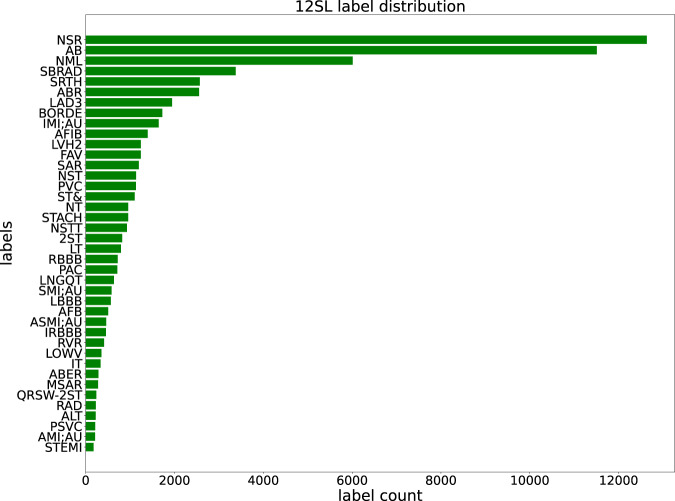
*PTB-XL* label distribution according to 12SL’s automatic diagnostic statements (showing the 40 most frequent statements out of overall 117 statements present in the whole dataset).

In Fig. 4, we visualize the label distribution according to the automatic 12SL diagnostic statements (column *statements_ext* in 12sl_statements.csv). The acronyms used in Fig. 4 are described in 12slv23ToSNOMED.csv. The distribution of statements over the full *PTB-XL* dataset covers 117 statements and therefore provides a rich source of information - in particular in comparison to the original labels provided within the *PTB-XL* dataset. In the Technical Validation Section, we provide a first quantitative comparison between both label sets based on SNOMED CT terms as common vocabulary.

## Technical Validation

The technical validation for the *PTB-XL* + dataset covers three different aspects. First, we assess the consistency of the different ECG features sets by comparing output distributions as well as comparisons on the level of individual samples. Second, we use the performance level of Random Forest classifiers trained on different feature sets on standard ECG prediction tasks^26^ as an indirect measure for the discriminative power of the different feature sets. Third, we investigate the correlation between the automatic 12SL ECG statements and the ECG statements provided within the *PTB-XL* dataset by cardiologists. Finally, we assess the performance of state-of-the-art deep learning models^26^ trained on the original *PTB-XL* labels and evaluated on 12SL-labels and vice-versa.

### ECG features: Consistency between different feature sets

**Fig. 5:**
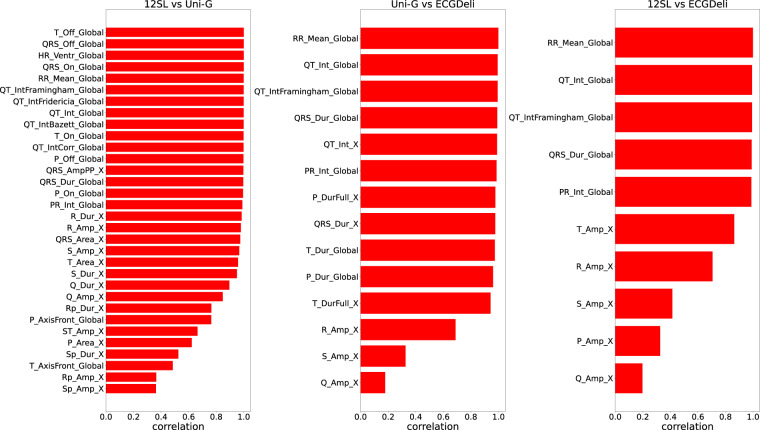
Feature comparison based on (Pearson) correlation coefficients (left: 12SL vs. Uni-G, center: Uni-G vs. ECGDeli, right: 12SL vs. ECGDeli).

In Fig. 5, we compare the different feature sets based on sample-wise Pearson correlation coefficients of those ECG features that are each contained in two of the feature sets under consideration, where we restrict ourselves for simplicity to continuous features. At this point, it is worth stressing again that this is to the best of our knowledge the first publicly available set which allows for a quantitative comparison between ECG features, in particular including those from two leading commercial providers. To simplify the presentation, we compute lead-specific correlation coefficients but only report average correlation coefficients across all 12 leads for lead-specific ECG features.

The left panel in Fig. 5 compares the two commercial algorithms 12SL and Uni-G and shows very good agreement among all common global features. Also most of the lead-specific standard amplitude and interval features show a good agreement with correlation coefficients above 0.9. The least agreement show features related to R’ and S’ (i.e., a second positive/negative wave after the R/S-wave), which are potentially more difficult to detect, and certainly are features for which some deviations might potentially also be due to different definitions. The center and the right panel of Fig. 5 show the comparison to the ECGDeli features. Again, one observes good agreement for the global features and many interval features, reasonable agreement for T and R amplitudes and least agreement for S, P and Q amplitudes.

### ECG features: Assessing the discriminative power of different feature sets

Following the evaluation protocol established in^26^, we train Random Forest classifiers on the different feature sets to assess their discriminative power, when used as input features for comprehensive ECG classification tasks. As the three feature sets are composed differently, we also consider training on feature subsets that two feature extraction algorithms have in common, which in principle allows for a direct comparison of the discriminative power of features extracted by different algorithms. We assess the performance on the set of seven multi-label prediction tasks put forward in^26^ and report the macro-average (across labels) of the respective areas under the receiver operating curves, henceforth referred to as macro AUC, on the *PTB-XL* test set. For reference, we also report the published performance scores of the xresnet1d101, a convolutional neural network operating on the raw waveform data^26^.

First of all, the results compiled in Table 2 reveal that all three feature sets are highly predictive, reaching mean macro AUC values of 0.889, 0.871 and 0.879 for Uni-G, 12SL and ECGDeli, respectively. On their entire respective feature sets (denoted as “full”), the Uni-G features are most discriminative. Interestingly, while the feature-based approaches fail to reach the CNN performance on comprehensive classification tasks (such as “all”), ECGDeli outperforms the CNN baseline in the rhythm category. This is in slight tension to the results from^27^, where the authors found that feature-based and raw-signal-based approaches lead to comparable performance across several diagnostic categories. We also provide results for models trained on the set of features shared by two feature sets (line 5–10 Table 2), which allows for a more direct comparison between the two feature sets. The results reveal that Glasgow and 12SL features have comparable quality but both are superior to the ECGDeli features (leaving aside the rhythm category).

**Table 2:** Classification performance on *PTB-XL* benchmarking tasks^26^ (macro AUC on the *PTB-XL* test set) achieved using different feature sets using different *PTB-XL* label (sub)sets as targets (all: all 71 statements, diag: 44 diagnostic statements, sub-diag: 23 aggregated, sub-diagnostic statements, super-diag: 5 aggregated, super-diagnostic statements, form: 19 form-related statements, rhythm: 12 rhythm-related statements).

Model/Features	all	diag	sub-diag.	super-diag.	form	rhythm
CNN/raw data^26^	0.925	0.937	0.929	0.928	0.896	0.957
RF/Uni-G(full)	**0.875**	**0.907**	**0.886**	0.921	**0.803**	0.945
RF/12SL(full)	0.856	0.906	0.878	**0.924**	0.794	0.870
RF/ECGDeli(full)	0.864	0.891	0.883	0.899	0.776	**0.964**
RF/Uni-G(Uni-G ∩ 12SL)	0.855	0.890	**0.889**	**0.923**	0.773	**0.881**
RF/12SL(Uni-G ∩ 12SL)	**0.866**	**0.892**	0.881	0.922	**0.796**	0.860
RF/Uni-G(Uni-G ∩ ECGDeli)	**0.863**	**0.902**	**0.888**	**0.916**	**0.769**	0.892
RF/ECGDeli(Uni-G ∩ ECGDeli)	0.855	0.898	0.863	0.902	0.753	**0.906**
RF/12SL(12SL ∩ ECGDeli)	**0.857**	**0.894**	0.877	**0.919**	**0.781**	0.872
RF/ECGDeli(12SL ∩ ECGDeli)	0.855	0.884	**0.889**	0.903	0.764	**0.902**

Best-performing feature-based approaches in each category are marked in bold face. Overall best-performing approaches are underlined.

### Automatic diagnostic statements: Agreement between 12SL and original *PTB-XL* labels

#### Descriptive analysis

We study the overlap between cardiologists’ annotations provided as part of the *PTB-XL* dataset and the automatic 12SL diagnostic statements. We use the provided mapping to SNOMED CT terms (12slv23ToSNOMED.csv and ptbxlMapToSNOMED.csv as described in Data Records) to obtain compatible label sets. We consider the set of SNOMED CT terms that are present in both label sets while only keeping informative terms, see the description in the Section Data Records. This leaves us with 94 SNOMED CT terms that can be directly compared across both label sets.

**Fig. 6:**
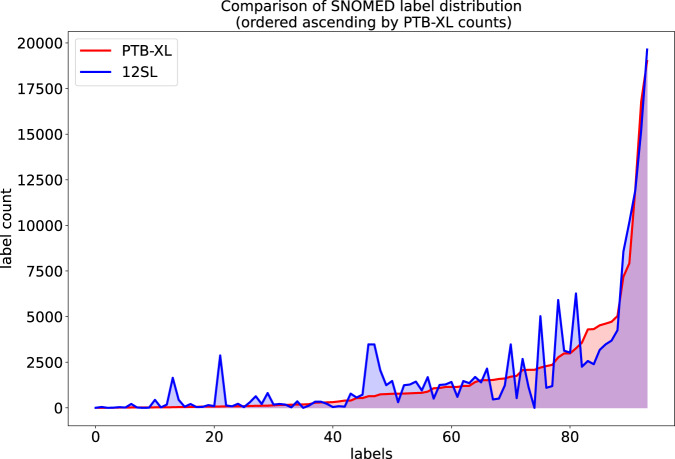
Visual comparison of the label distribution for 12SL vs. original *PTB-XL* after mapping to SNOMED CT. On the x-axis we show the SNOMED CT labels ordered by ascending counts in the *PTB-XL* label set.

First, we visually compare the label distributions in Fig. 6, where we show the label occurrence for the common SNOMED identifiers in the 12SL vs. the original *PTB-XL* label set after mapping to SNOMED CT (ordered by occurrence in *PTB-XL*), which shows a rough overlap in terms of label distributions.

To investigate this in more detail on the per-sample level, we compute the Matthews Correlation Coefficient (MCC)^28^ between the binarized scores obtained from selecting the non-zero values of the continuous scores. The result of this analysis is listed in Tables 3, 4. The median of the correlation across all terms is 0.45. In particular, we find good agreement for atrial fibrillation, complete bundle branch blocks, sinus tachycardia (all with MCC above 0.8), which aligns with cardiologists’ knowledge as these conditions are rather clearly identifiable from a 12-lead ECG. On the other hand, there is also a range of statements, including myocardial infarctions with specific localization, with essentially no agreement. In any case, these findings provide valuable hints for future investigations of the label quality of the *PTB-XL* annotations and the 12SL statements.

**Table 3:** Correlation between automatic 12SL and cardiologists’ labels on *PTB-XL* (listing only samples where both counts exceed 50).

SNOMED CT Concept identifier	MCC	Count PTB-XL	Count 12SL	description
313217	0.89	1514	1396	Atrial fibrillation
4267892	0.88	536	566	Complete left bundle branch block
4007310	0.88	826	957	Sinus tachycardia
4304202	0.83	294	336	Rhythm from artificial pacing
4088337	0.82	541	721	Complete right bundle branch block
4145998	0.79	1143	1420	ECG: premature ventricular contractions
4089462	0.78	1197	1469	Ventricular premature complex
4145513	0.75	18978	19634	ECG: sinus rhythm
314059	0.74	1658	1229	Right bundle branch block
4091901	0.69	1513	2151	Aberrant premature complexes
4065279	0.67	9514	8587	ECG normal
313791	0.64	3659	2258	Bundle branch block
320536	0.64	12864	11904	Electrocardiogram abnormal
4185932	0.63	6973	5462	Ischemic heart disease
4027255	0.63	8002	6483	Structural disorder of heart
4329847	0.63	5469	4174	Myocardial infarction
4064609	0.61	5469	4338	EKG: myocardial infarction
4166844	0.61	4393	2568	Intraventricular conduction defect
4247796	0.61	3238	2304	Inferior myocardial infarction on electrocardiogram
4166245	0.59	5699	3511	Disorder of cardiac ventricle
4064614	0.58	2332	1099	EKG: left bundle branch block
4064457	0.58	4412	2395	EKG: heart block
320425	0.57	4822	3687	Heart block
314665	0.56	73	151	Atrial flutter
316998	0.56	2404	1193	Left bundle branch block
4068155	0.55	2207	5031	Atrial arrhythmia
44784217	0.54	8011	10228	Cardiac arrhythmia
314379	0.52	793	1240	First degree atrioventricular block
4248028	0.52	2766	5914	Supraventricular arrhythmia
4111570	0.51	807	1280	Partial atrioventricular block
316135	0.49	823	1438	Atrioventricular block
4088338	0.49	1118	508	Incomplete right bundle branch block
3655971	0.49	3379	6660	Atrial cardiopathy
4217221	0.48	772	1473	Nodal rhythm disorder
43020843	0.47	915	1693	Disorder of right atrium
4008580	0.47	82	85	Ventricular bigeminy
4295336	0.46	1623	507	Left anterior fascicular block
4064610	0.45	2357	566	Anteroseptal infarction on electrocardiogram
43021828	0.44	99	132	Right atrial enlargement
4184746	0.44	2132	1256	Left ventricular hypertrophy
442982	0.44	2251	1378	Left ventricular abnormality

**Table 4:** Correlation between automatic 12SL and cardiologists’ labels on *PTB-XL* (continued).

SNOMED CT Concept identifier	MCC	Count PBT-XL	Count 12SL	description
37017193	0.43	1194	602	EKG: Incomplete right bundle branch block
4034164	0.43	1797	533	Monofascicular block
4145489	0.42	2254	1299	Ventricular hypertrophy
4169095	0.38	637	3492	Bradycardia
4171683	0.38	637	3476	Sinus bradycardia
4115173	0.36	398	780	Atrial premature complex
4064346	0.36	2467	1772	EKG myocardial ischemia
4186397	0.36	2467	1772	Myocardial ischemia
4184348	0.32	288	146	Anterolateral infarction by electrocardiogram
4139185	0.3	208	474	EKG: anterior ischemia
312327	0.29	150	178	Acute myocardial infarction
4137208	0.29	397	541	EKG: inferior ischemia
4184762	0.28	1815	3478	EKG ST segment changes
4171193	0.28	82	224	Idioventricular rhythm
4132088	0.27	174	178	Acute heart disease
4185572	0.22	102	305	Ventricular arrhythmia
4327859	0.2	767	2062	Nonspecific ST-T abnormality on electrocardiogram
438170	0.2	55	89	Acute myocardial infarction of inferior wall
43022066	0.2	426	485	Left atrial enlargement
4064350	0.19	679	190	Lateral infarction on electrocardiogram
4008859	0.18	117	636	Prolonged QT interval
4065390	0.16	2070	2747	EKG: T wave abnormal
44784220	0.14	787	311	Non-specific intraventricular conduction delay
4231591	0.12	126	60	Right ventricular hypertrophy
4263712	0.12	323	288	Subendocardial ischemia
4088499	0.12	182	356	Low QRS voltages
4137879	0.1	140	1293	EKG: lateral ischemia
314666	0.08	84	3999	Old myocardial infarction
4121467	0.05	54	2256	Old inferior myocardial infarction
4064461	0.05	871	1243	ECG: ST interval abnormal
4088336	0.04	77	94	Incomplete left bundle branch block
4119949	0.04	55	618	Old anterior myocardial infarction
4109365	0.0	398	67	Premature atrial contraction
4065287	0.0	157	214	EKG: supraventricular arrhythmia
4180609	−0.02	353	618	Anterior myocardial infarction on electrocardiogram

#### Model training

To assess the quality of the 12SL labels, we conducted a series of model training experiments, the results of which are shown in Table 5. First, we used the original 12SL labels and trained an xresnet1d50 classification model, which is a modern convolutional neural network, which was found to perform well on *PTB-XL* across various prediction tasks^26^. We used the first eight stratified folds (training set) from *PTB-XL* for training, the ninth fold (validation set) for model selection via early stopping and report the macro AUC on the tenth fold (test set). Further, we discarded labels, that do not occur at least once in all of the before-mentioned splits, leaving us with 109 labels. The xresnet1d50 reaches a macro AUC of 0.956 demonstrating that the full input signals are very discriminative for the prediction of the 12SL labels.

**Table 5:** Model performance for different label sets and train/test scenarios.

Label-set	Train labels	Test labels	macro AUC
12SL original	12SL	12 SL	0.956
SNOMED CT	12SL	12 SL	0.939
SNOMED CT	PTB-XL	PTB-XL	0.912
SNOMED CT	12SL	PTB-XL	0.867
SNOMED CT	PTB-XL	12SL	0.867

Here, PTB-XL refers to the original labels provided in *PTB-XL* (after mapping to SNOMED CT).

To investigate the comparability of the 12SL labels with the original *PTB-XL* labels, we use the provided mapping to SNOMED CT labels (up-propagated in the label hierarchy) that was described above. After removing uninformative SNOMED CT labels close to the SNOMED CT root node (and SNOMED CT labels that show perfect correlation to other labels on both datasets) and discarding all those SNOMED CT labels that did not appear in each split, we reduced the label set to 168 SNOMED CT codes. Following the same procedure as described above, we report again the macro AUC on the test set in Table 5. In addition, we also report the results of cross-evaluation of models trained on the 12SL SNOMED CT labels and evaluated on the *PTB-XL* SNOMED CT labels and vice versa. Models trained and evaluated on labels stemming from the same original source show a high predictive performance (0.939 vs. 0.912 for 12SL vs. original *PTB-XL* labels). The cross-evaluation results are in both cases considerably weaker but very similar (0.867 in both cases). The precise understanding of this discrepancy is an interesting direction for future research.

## Usage Notes

We structure the usage instructions according to the different components provided in the dataset:

- **ECG-features** are provided as csv-files, which can be read by any standard software.
- **Median beats and fiducial points** are provided in PhysioNet’s wfdb format^25^, which can be conveniently processed using toolkits in C, MATLAB and Python.
- **Automatic diagnostic statements** are again provided as csv-files for easy accessibility.

For the user’s convenience, we release the classifier training code^19^ for the experiments presented in the Technical Validation Section. This should provide a good starting point for own explorations of the dataset. We believe that the availability of the additional features provided will significantly enhance the usability of the *PTB-XL* dataset due to the ability to train ML models on features and combinations of raw data and features, to look into the quality of features from different feature sets and into the strengths and weaknesses of diagnostic statements provided by state-of-the-art ECG analysis software.

## Data Availability

The ECG features directly correspond to the outputs of the respective algorithms up to minor harmonization. We provide code to apply the predefined SNOMED CT mappings to the labels in the dataset (apply_snomed_mapping.py released as part of the data repository^24^). Links to code samples facilitating the usage of the dataset are described under Usage Notes and are released in a dedicated code repository^19^.
